# ECP-induced Apoptosis: How Noninflammatory Cell Death Counterbalances Ischemia/Reperfusion Injury

**DOI:** 10.1097/TXD.0000000000001816

**Published:** 2025-09-02

**Authors:** Julia Stępień, Elke Eggenhofer

**Affiliations:** 1 Department of Surgery, University Hospital Regensburg, Regensburg, Germany

## Abstract

Extracorporeal photopheresis (ECP) is a therapeutic procedure that is increasingly recognized for its efficacy in treating immune-mediated diseases, including transplant rejection. Its main mechanism is ex vivo apoptosis induction in leukocytes from patients by incubation with 8-methoxypsoralen and irradiation with ultraviolet A light. The process involves DNA cross-linking, which leads to a cascade of events within the cell and ultimately to apoptosis induction. Although ECP has been used for almost 40 y, there remain many questions about its immunological mechanisms and therapeutic potential. Here, we review current knowledge about mechanisms of apoptosis induction in subsets of peripheral blood mononuclear cells and interactions of apoptotic leukocytes with immune cells. We also highlight the challenges of reproducibly inducing cell death in a clinical manufacturing procedure and propose innovative ways to improve and quality-control ECP photopheresates.

The relationship between apoptotic cell death and immunosuppression is already well recognized, but the diverse effects of apoptotic cells in different cell types are still being investigated. A key step in the therapeutic mechanism of extracorporeal photopheresis (ECP) is the recognition and phagocytosis of apoptotic leukocytes by dendritic cells (DCs) and macrophages, driving them to a tolerogenic state.^1–3^ In turn, these regulatory myeloid antigen-presenting cells promote the development of antigen-specific regulatory T-cell responses.^4,5^ ECP-induced apoptotic cells also likely influence other innate immune cells, including natural killer (NK) cells and γδ T cells, to change cytokine expression profiles^6^ and induce regenerative phenotypes.^7–9^ Because control of innate immunity, immune regulation, and tissue repair are tightly linked processes, we hypothesize that ECP may have beneficial clinical effects beyond controlling tissue inflammation.^10–12^ In particular, we hypothesize that apoptotic cells from photopheresates condition their local environment by releasing regenerative factors during the early stages of cell death that stimulate tissue regeneration and repair.^12^ Although not currently approved for this indication, these proresolving effects of ECP may be useful in treating ischemia/reperfusion injury (IRI) or alloimmune tissue damage after solid organ transplantation.^13^

A first wave of inflammation after organ transplantation is triggered by danger associated molecular patterns (DAMPs) induced by IRI.^14^ DAMPs activate cells of the innate immune system and T cells,^15^ especially interleukin (IL)-17-producing γδ T cells,^16^ leading to tissue damage through neutrophil recruitment, inducing parenchymal cell necrosis and secondary release of DAMPs.^17^ We hypothesize that release of anti-inflammatory factors from apoptotic cells could reduce such tissue damage by decreasing activation of γδ T cells in early phases after transplantation. However, to our knowledge, nothing has yet been published regarding the ECP-induced effects on γδ T cells.

## MECHANISM OF APOPTOSIS INDUCTION

During ECP therapy, apoptosis is induced in leucocytes collected by apheresis through ex vivo exposure to ultraviolet A (UVA) irradiation (λ = 315–400 nm) in the presence of 8-methoxypsoralen (8-MOP; methoxysoralen; UVADEX). The drug is a synthetic photoreactive substance belonging to the psoralen group of compounds. Psoralens are natural, plant-derived compounds that penetrate phospholipid cellular membranes to intercalate between the pyrimidine bases of DNA. In the absence of UV light, they remain photochemically inert and are removed from cells within 24 h, without affecting most cell types, even at relatively high concentrations.^18,19^

Activation of 8-MOP is triggered by UVA light, leading to photochemical reactions that result in the formation of DNA adducts. These adducts inhibit replication by interstrand cross-linking of the DNA.^18,20^ Formation of interstrand cross-linkings leads to double-strand DNA breaks (DSBs) as a natural DNA repair mechanism of the cell.^21^ At high densities, these are lethal to cells,^20,22,23^ as they are far-reaching genomic lesions leading to genomic instability.^24^ Even in small numbers, DSBs constitute a serious threat to cell survival by activation of DNA damage signaling,^25^ and their defective repair may lead to major cellular changes, resulting in cell death.^24^ DSBs are thought to be the main stimulus for apoptosis induction in psoralen-triggered cell death^26^ because unrepaired DSBs initiate a signaling cascade that eventually leads to apoptosis. The process begins with cell cycle arrest and restraining the ability of a cell to repair DNA damage, which leads to the release of proapoptotic molecules and mitochondrial membrane permeabilization. The Bax protein is a central effector in this process, causing mitochondrial damage and triggering the intrinsic pathway of apoptosis.^27^

## KINETICS OF APOPTOSIS AFTER ECP

ECP treatment affects leucocyte subsets differently; however, differences in apoptosis induction can also be observed between cells of the same cell type.^28^ One of the reasons might be differences in the sensitivity of cells to 8-MOP/UVA treatment.^9^ After treatment with 8-MOP and exposure to UVA light, a small fraction of cells become apoptotic very rapidly, meaning that between the end of leukapheresis collection and reinfusion, apoptosis can be detected through the presence of phosphatidylserine at the outer surface of cell membranes.^29–32^ This early wave of apoptosis is thought to be connected with mitochondrial membrane rupture. Later, a second wave of ECP-treated cells goes into cell cycle arrest^33–35^ and, from 20 h onward, most cells undergo cell death. At this stage, an increase in the death receptor CD95 (Fas) can be detected.^36,37^ Induction of the CD95 pathway initiates apoptosis by activation of the caspases, which belong to the family of apoptotic proteases. Elevated caspase activity has been observed 24–48 h after ECP treatment.^9^ Although many studies have looked at apoptosis induction by ECP, it remains unclear how exactly the different mechanisms synergize to initiate apoptosis over time.

## CELL TYPE–SPECIFIC SENSITIVITY TO APOPTOSIS INDUCTION

Lymphocytes are more sensitive to 8-MOP/UVA-induced apoptosis than monocytes. T cells appear to be most susceptible to apoptosis, particularly when activated.^38^ Apoptosis of T cells is important for the immunomodulatory action of ECP owing to their release of anti-inflammatory factors; notably, apoptotic T cells suppress activated macrophages through secretion of transforming growth factor-β and other soluble mediators.^39^

Monocytes prove to be more resistant to 8-MOP/UVA-induced apoptosis than T cells immediately after ECP treatment; however, they also have a high death rate after longer in vitro culture. Interestingly, preapoptotic monocytes retain some of their functions, for instance, ECP stimulates their migratory function, and they remain capable of endocytosis.^40^ It has been suggested that a proportion of ECP-treated monocytes can differentiate into monocyte-derived DCs.^41^ Some sources attribute this effect to mechanical forces, flow in an ECP device, adherence to plastic surfaces, and/or involvement of platelets.^42–45^ Such ECP-induced, monocyte-derived DCs migrate after reperfusion to the lungs, then subsequently to the liver and secondary lymphoid organs,^46^ where they interact with myeloid antigen-presenting cells. In the spleen, it is hypothesized that apoptotic monocyte-derived DCs from photopheresates could influence immature DCs. This interaction leads to suppression of their maturation into stimulatory DCs. Through uptake of apoptotic cells, DCs convert to a tolerogenic phenotype, releasing transforming growth factor-β and inducing FoxP3^+^ regulatory T cells.^11,47,48^ Although there is little evidence described in the literature about trafficking ECP-treated leukocytes in the body, new findings suggest the ability of such cells to migrate selectively to inflammation sites.^3^

ECP seems to affect NK cells. In vitro studies have shown that NK cells are one of the first leucocyte subsets to undergo apoptosis after ECP.^49,50^ Moreover, 8-MOP/UVA treatment impairs their cytotoxic function. One of the hallmarks of NK-cell activation is degranulation—the secretion of lytic granule components onto the target cell surface, which can be detected with the CD107a marker. A significant decrease in this marker was observed in irradiated NK cells after 24 h of incubation in cell culture.^7,51^ The level of its reduction has been correlated to the hematocrit (HCT) level in the patient sample,^51^ which is strongly correlated to successful apoptosis induction. Moreover, some studies suggest that ECP supports the expansion and development of the regulatory subset of NK cells in response to elevated levels of interleukin-15 resulting from apoptotic cell death of alloreactive T cells.^7,52^

Research on the sensitivity of B cells to ECP-induced apoptosis is inconsistent and requires further study. However, there is evidence of apoptosis induction in this type of cell.^49,50^

## APOPTOSIS AND IMMUNE CELL INTERPLAY

Dynamic clearance of reinfused apoptotic cells by phagocytes is one of the first steps of ECP treatment. Apoptotic cells possess immunomodulatory potential on many different levels, which are supported by different mechanisms; however, if they are not cleared in the right time frame, they become secondarily necrotic, which leads to the release of proinflammatory molecules.^53^

Because numerous processes happen in a cell when apoptosis is induced, they can affect many different cell types and the environment. The most widely described are signals directed to macrophages, which include “find-me” and “eat-me” signals. The “find-me” signals in the form of released soluble factors that act as attractants for phagocytes to migrate toward apoptotic cells, and subsequently prepare them for engulfing cells by modulating their cytoskeleton, enhancing the expression of engulfment receptors, and influencing the phagocytic machinery.^54^ The “eat-me” signals are exposed on the surface of a dying cell and are recognized by phagocytic receptors.^55^ Efferocytosis of apoptotic cells by macrophages leads to release of interleukin-10,^56^ one of the most important factors in resolving inflammation, as well as an inhibitor of other inflammatory cell recruitment.^10,55^ By this mechanism, tolerogenic macrophages are induced in the ECP process.^56,57^

The apoptotic cell death pathway is highly regulated and is able to induce immunosuppression not only by direct interactions with phagocytes but also by regulated release of metabolites. Over 100 metabolites can be released by a cell in a regulated manner in the early stages of apoptosis.^6^ Released metabolites differ between cell types, but a few key metabolites are common to different cell types and trigger apoptosis.^12^ It is hypothesized that released metabolites contribute to a regenerative tissue environment.^6,12,55^

Another immunomodulatory effect of ECP is the initiation of monocyte-to-dendritic cell differentiation,^58,59^ and DC maturation. These aspects are thought to be an important step in the therapy. It is necessary to note that here we describe 2 different mechanisms. One refers to monocytes in the ECP product, with previously induced apoptosis, and was described in more detail in the Cell Type–specific Sensitivity to Apoptosis Induction section. The other mechanism concerns immature DCs residing in secondary lymphoid organs of the patient. Induction of DC differentiation by ECP^1,2,58,59^ in apoptotic monocytes provides immunosuppressive properties that reduce systemic inflammation.^56^ After reperfusion, immature DCs are primed by apoptotic DCs. In this context, priming involves the capture of apoptotic cells with self-antigen, which induces maturation of DCs into a tolerogenic phenotype, that in turn induces regulatory T cells in vivo.^1,2,60,61^

## DETERMINANTS OF APOPTOSIS INDUCTION

**FIGURE 1.**
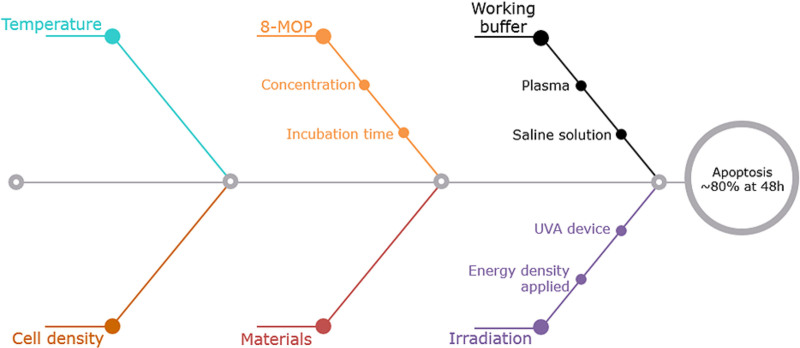
Technical aspects of ECP therapy influencing apoptosis induction. ECP, extracorporeal photopheresis.

Ex vivo 8-MOP/UVA-induced apoptosis is influenced by many factors (Figure 1). The rate and extent of 8-MOP-induced apoptosis are dependent upon drug doses and incubation time. The dose of 8-MOP must be adjusted to the volume and cell density of leucapheresates to achieve ~80% T-cell apoptosis by 48 h posttreatment. Nonetheless, even if the drug is in excess, free 8-MOP is photo-inactivated, so there are no adverse reactions in patients.^62^ This natural inactivation of 8-MOP ensures that there is no risk of inducing apoptosis in cells other than those that have been treated.^62^ Incubation times differ between ECP providers, mostly for organizational reasons; however, longer (up to 30 min) incubation results in higher rates of apoptotic cells.^49^ This effect is due to the diffusion of 8-MOP into the cells, which takes minutes, although reaching equilibrium might take up to 30 min.^63^ Other factors include a dose of irradiation and the surface of a set material, which might be more or less susceptible to adhesion of 8-MOP.^63^ However, these parameters of ECP are currently well established, giving medical centers the choice not to jeopardize treatment success. A more complex problem with regard to apoptosis induction is the choice of buffer in which the cells are suspended after apheresis and incubated with the drug, where plasma and buffered saline solutions are commonly used. Plasma reduces cellular uptake of 8-MOP owing to plasma proteins binding to the drug; however, nonspecific proteins also reduce 8-MOP adhering to plastic surfaces. Incubating leucocytes with the drug in saline solution results in higher rates of apoptosis induction.^63^

It is important to remember that leucocytes collected during the ECP procedure are not in the form of a pure cell suspension of immune cells; there is always a contribution of erythrocytes (red blood cells). Therefore, the efficiency of apoptosis induction is highly dependent on the HCT level. If HCT is >4%, the ability to induce apoptosis is compromised because of UVA shading by red blood cells,^63^ so irradiation times must be adjusted to achieve optimal apoptosis rates.

Evaluating the apoptosis rates of photopheresates is essential for the assessment and potential optimization of the setup. Apoptosis staining and measurement by flow cytometry present a time-efficient, low-cost method with stable results.^64^ This method is based on staining with Annexin V—protein binding to phosphatidylserine (PS)—and propidium iodide or 7-AAD—dyes binding to DNA of cells with a ruptured membrane. Readout of the staining with flow cytometry allows us to distinguish alive, apoptotic, and necrotic cells. For a more insightful analysis of cell types, lineage markers, such as CD45, CD3, and CD14 antibodies, should be used.

## DEVELOPMENT PROSPECTS

ECP is widely used, safe, and brings undeniable benefits to selected subsets of transplant recipients,^32,65^ but there are still many questions about its immunological mechanisms of action.^6,15,32,56,65–67^ Advances in genomics, proteomics, and metabolomics of apoptotic cell secretomes could uncover specific pathways that are activated during ECP, allowing the design of therapeutic strategies to maximize its clinical impact. We believe there is great potential in individualizing ECP treatment according to patient factors. We urgently need new biomarkers to assess the pharmacodynamics of apoptotic cells after reinfusion in patients, because this would allow us to monitor whether induced responses are functioning optimally.

There is growing interest in combining ECP with other immunotherapies or treatments to enhance its therapeutic effects. Research into how ECP-induced apoptosis interacts with other immune-modulating therapies could open new avenues for combination treatments in disorders driven by overactivation of the immune system. In particular, one promising area is early post–organ transplantation in patients who receive marginal organs or develop severe IRI. Here, we hypothesize that ECP could help prevent immune responses against grafts with prior injury or reduced regenerative capacity.
